# Human Cytomegalovirus DNA Quantification and Gene Expression in Gliomas of Different Grades

**DOI:** 10.1371/journal.pone.0159604

**Published:** 2016-07-26

**Authors:** Lucas Matheus Stangherlin, Fabiane Lucy Ferreira Castro, Raphael Salles Scortegagna Medeiros, Juliana Mariotti Guerra, Lidia Midori Kimura, Neuza Kazumi Shirata, Suely Nonogaki, Claudia Januário dos Santos, Maria Cristina Carlan Silva

**Affiliations:** 1 Laboratório de Biologia Molecular de Patógenos, Virologia Molecular, Centro de Ciências Naturais e Humanas, Universidade Federal do ABC, São Paulo, Brazil; 2 Departamento de Anatomia Patológica do Hospital das Clínicas da Faculdade Medicina da Universidade de São Paulo, São Paulo, Brazil; 3 Núcleo de Patologia Quantitativa (NPQ) do Centro de Patologia do Instituto Adolfo Lutz (IAL), São Paulo, Brazil; University of St Andrews, UNITED KINGDOM

## Abstract

Gliomas are the most common type of primary brain tumors. The most aggressive type, Glioblastoma multiforme (GBM), is one of the deadliest human diseases, with an average survival at diagnosis of about 1 year. Previous evidence suggests a link between human cytomegalovirus (HCMV) and gliomas. HCMV has been shown to be present in these tumors and several viral proteins can have oncogenic properties in glioma cells. Here we have investigated the presence of HCMV DNA, RNA and proteins in fifty-two gliomas of different grades of malignancy. The UL83 viral region, the early beta 2.7 RNA and viral protein were detected in 73%, 36% and 57% by qPCR, ISH and IHC, respectively. Positivity of the viral targets and viral load was independent of tumor type or grade suggesting no correlation between viral presence and tumor progression. Our results demonstrate high prevalence of the virus in gliomas from Brazilian patients, contributing to a better understanding of the association between HCMV infection and gliomas worldwide and supporting further investigations of the virus oncomodulatory properties.

## Introduction

Gliomas are the most common intra axial brain tumors in adults, representing 40 to 60% of primary central nervous system (CNS) tumors[1], with an annual incidence of 5/100,000 individuals. According to the World Health Organization (WHO) classification of CNS tumors, gliomas are divided in ependymomas, astrocytomas, oligodendrogliomas, and mixed oligoastrocytomas. This universal classification system considers the morphology of tumor cells, tissue architecture, and immunohistological marker profiles [2]. Based on malignant tumor behavior astrocytomas are divided in pilocytic (AST I), diffuse (AST II), anaplastic (AST III) and glioblastoma multiforme (GBM) (AST IV). Oligodendrogliomas are divided in grade II (ODG II) or III (ODG III) [3].

Astrocytoma pilocytic is the least aggressive and may be cured by surgery, while astrocytomas grade II, III and IV are more aggressive. Glioblastoma multiforme is the deadliest type and accounts for half of the cases of gliomas [4]. The median survival for GBM patients is 12–18 months [5].

Oligodendroglial tumors are less common. They comprise approximately 5% of the gliomas and the affected patients have a better prognosis than those with astrocytic neoplasms [6].

The etiological factors for gliomas are unknown. However, genetic inherited abnormalities are credited as the cause of a small portion of the cases, and environmental factors such as exposure to ionizing radiation are postulated to be risk factors [7].

It is recognized that infectious agents, mainly viruses, are etiological agents of 10%-15% of human cancers worldwide. The so-called tumor viruses, such as Human Papilloma Virus (HPV) and Epstein-Barr Virus (EBV), encode gene products that can induce cellular transformation under certain circumstances [8]. Human Cytomegalovirus (HCMV) is a widespread pathogen present in 70–90% of the population worldwide[9].HCMV DNA, RNA, and antigens have been detected in a variety of tumor tissues, such as colorectal cancer [10,11], lung carcinoma [12], prostatic intraepithelial neoplastic and prostatic carcinoma [13], hepatocellular carcinoma [14], medulloblastomas[15], neuroblastomas[16], salivary gland cancer [17,18] and gliomas [19,20].

The relationship of HCMV with cancer, especially with regard to gliomas, is a topic of great debate. The presence of the virus in gliomas was initially demonstrated by Cobbs et al[21]and this finding was followed by various studies that investigated the existence of the virus in these tumors by different techniques (S1 Table).

Most studies show a positive association between HCMV and gliomas [1,20–39]. Nevertheless, there are investigations that could not confirm the presence of the virus in the tumors and controversy in the field persists [19,40–47]. In addition, few studies investigated the correlation between expression of viral genes and proteins in gliomas with tumor malignancy [20,30,36,37,48].

Here, we determined the presence of HCMV in gliomas from Brazilian patients. Our results confirm the presence of the virus in tumors of different grades and suggest that there is no association between the presence of viral RNA, protein and DNA copy number, with tumor type or grade.

## Materials and Methods

### Tissue samples

A total of fifty-two paraffin-embedded tumor brain samples were obtained from the Department of Pathology—Hospital das Clínicas–Faculdade de Medicina da Universidade de São Paulo—Brazil.

Specimen collection and study procedures were approved by the CAPPesq: Research Ethics Committee from Hospital das Clínicas da Faculdade de Medicina da Universidade de São Paulo (approval number 466/2012).

The pathologist re-examined the cases to confirm diagnosis and the histological classification of tumors was based on the World Health Organization criteria.

In total, tissues from 26 astrocytomas (AST grades I-III), 10 glioblastoma multiformes (AST grade IV/GBM), 16 oligodendrogliomas (ODG grades II and III) and 13 non-tumoral brain specimens (NTB) from epileptic patients were evaluated for the presence of HCMV.

### *In situ* Hybridization

Paraffin sections of 5 μm thickness were deparaffinized in xylol and ethanol in decreasing degree to hydration. Samples were incubated with proteinase K (15 μg/mL) in 0.05M Tris/HClbuffer pH 7.6 for 20 min at 37°C and washed in water for 10 minutes. Sections were dehydrated in 95% followed by 99% ethanol for 3 minutes each and air-dried.

Ten microliters of a fluorescein-labeled probe for detection of the highly abundant beta 2.7 early HCMV transcript (Leica Microsystems, NCL-CMV, Newcastle upon Tyne, UK) (McSharry et al, 2003), was placed on the sections and then sealed. Sections containing the probe were incubated overnight at 37°C in Hybridizer (Dako, S2450, Glostrup, Denmark), and detection was performed following manufacturer´s instructions (Novocastra*In situ* Hybridization Detection Kit, Leica Microsystems, NCL-ISH-D, Newcastle upon Tyne, UK). Slides were incubated with blocking solution followed by rabbit F(ab´) anti-FITC conjugated to alkaline phosphatase and developed with BCIP (5-bromo-4-chloro-3-indolyl phosphatase) and NBT (nitro blue tetrazolium) with 1M levamisole. Slides were washed with TBS (Tris-buffered saline) three times of 5 min in each step. Mayer´s hematoxylinand eosin (HE) were used to counterstain and slides were mounted with aqueous mountant (Merck, Aquatex, Darmstadt, Germany) and analyzed.

Predominantly cytoplasmic and nuclear purple staining was considered positive. Positive controls consisted of lung, liver and colon tissues from patients, naturally infected with HCMV, previously confirmed by PCR and immunohistochemistry. RNA integrity was confirmed using a probe labeled with digoxigenin for polyadenilated mRNA (ZytoVision Probe T-1118-100 and developed with T-1063-40). Negative control was performed by omission of the probe.

### Immunohistochemistry

Paraffin sections of 3 μm thickness were deparaffinized in xylol and ethanol in decreasing degree to hydration. Antigen retrieval was performed in 0.01M sodium citrate buffer, pH 6.0, in a stainless steel pressure cooker for three minutes. After antigen retrieval, endogenous peroxidase blocking was performed with 6% aqueous hydrogen peroxide solution. Slides were washed in tap and distilled water and phosphate buffered saline (PBS). Histological sections were incubated overnight at 4°C with the anti-cytomegalovirus antibody, clone CMV01 (Abcam ab75116). The antigen-antibody binding was amplified with polymer conjugated to antibodies and peroxidase Expose (Mouse specific HRP/DAB detection IHC Kit, Abcam ab94710, Cambridge, MA, EUA). Slides were washed with PBS three times in each step. Sections were developed with diaminobenzidine substrate (Sigma, D5637, St Louis, MO, USA) and counterstained with Harris´ hematoxylin and eosin (HE). Nuclear golden brown was considered positive. Positive controls consisted of HCMV infected lung, liver and colon. Negative control was performed by omission of the antibody.

### Scoring of immunohistochemistry and *in situ* Hybridization

Immunohistochemistry and *in situ* hybridization were examined by the pathologist, for every sample was given a score based on the intensity of the staining (no staining = 0, low staining = 1, moderated staining = 2, strong staining = 3) and the percentage of positive cells (range 1–4: 25% increments). The IHC and ISH scores from 0 to 12, were calculated by determining the product of staining intensity (0–3) and positive stained cells (1–4). Samples with IHC and ISH scores equal or greater than 4 were considered positive.

### DNA extraction and Quantitative Real Time PCR

DNA was purified from paraffin-embedded tissues using RecoverAll™ Total Nucleic Acid Isolation Kit for FFPE (Life Technologies)according to the manufacturer’s instructions.

The quantitative Real Time PCR (qPCR) was performed using primers for UL83 viral region (forward 5’-CCC AGG TGT GTC GGT ACT CA– 3’; reverse 5’- CCA CCT TCA CCA GCC AGT ATC -3'), designed using PrimerExpress software (Applied Biosystems). The reactions were carried out in a *StepOne Plus* equipment (Applied Biosystems) in final volume of 13 μl containing 4 μl of DNA; SYBER Green 1x (Applied Biosystems) and 40 μM of each primer. The PCR conditions were as follows: 95°C for 10 min, 40 cycles of 95°C for 15 s, 60°C for 1 min. Data were collected and analyzed using the*Step One Software* v. 2.2 (Applied Biosystems). As a positive control for cellular DNA, primers for the Glyceraldehyde 3-phosphate dehydrogenase gene (GAPDH) gene were used (forward 5’—ACC CAC TCC TCC ACC TTT GAC—3’; reverse 5’—CTG TTG CTG TAG CCA AAT TCG T—3’). During HCMV detection no positive control was used to minimize contamination and water was used as a negative control after every three samples.

The limit of detection of viral DNA was determined using a standard curve (R² = 0,998) made by amplification of the UL83 region in 10-fold serial dilutions of HCMV FIX-BAC (HCMV FIX genome cloned as a Bacterial Artificial Chromosome), added to a known concentration of cellular DNA. The amount of viral DNA in the samples was determined using a standard curve made by amplification of the GAPDH cellular gene (R² = 0,999), as well as the UL83 standard curve. Viral load was calculated as the natural logarithm of the ratio between HCMV copy number/cell number.

### Statistical analysis

The Kruskal-Wallis statistical analysis was performed for IHC/ISH scores and qPCR viral load (p-values are shown in each respective figure). A P value < 0.05 was considered statistically significant.

## Results

### Detection of HCMV DNA, RNA and protein

**Fig 1:**
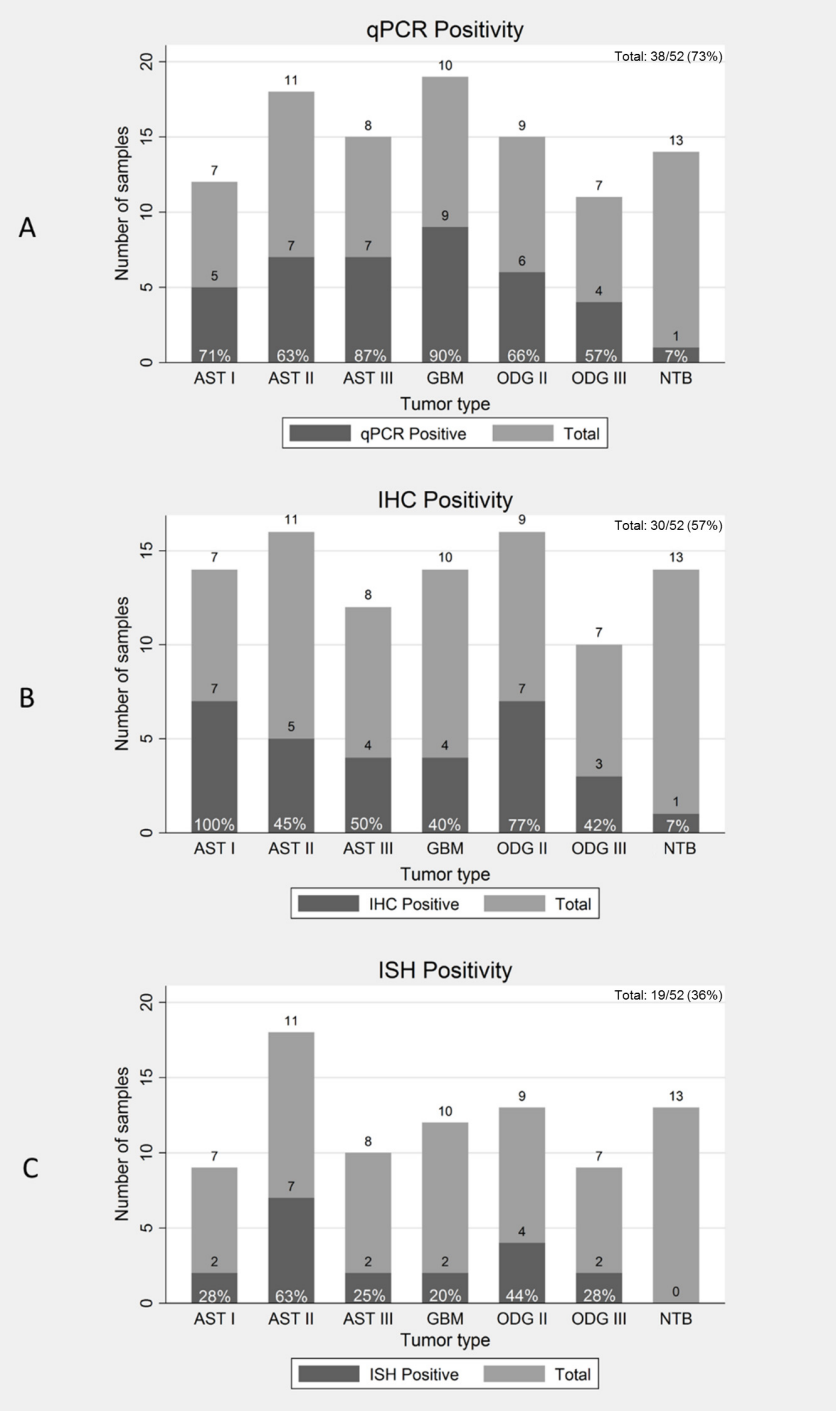
Human Cytomegalovirus positivity in gliomas. Total samples (Light gray), positive samples (Dark gray) and frequency of positivity are shown for astrocytomas (AST I, AST II, AST III and GBM), oligodendrogliomas (ODG II and ODG III) and non-tumoral brain (NTB) by qPCR (1A), IHC (1B)and ISH (1C).

To determine the presence of DNA in the tumor samples we performed qPCR using primers within the region of the viral genome encoding the UL83 (pp65) gene. The assay was previously optimized in our laboratory to detect low levels of viral DNA in GBM samples [32].The limit of detection, as calculated in a UL83 standard curve, was 1 copy of viral DNA per 500ng of total DNA (4 copies per 10^4^ cells). The UL83 region was detected in 38 of 52 (73%) of total tumors and in one non-tumoral brain sample (Fig 1A and S2 Table).

**Fig 2:**
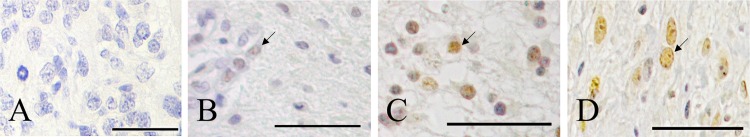
Immunohistochemical staining of HCMV in gliomas. (A)Negative GBM. (B) Positive GBM, score 4. (C) Positive AST II, score 8. (D) Positive control (HCMV infected colon). Arrows indicate HCMV nuclear protein expression in neoplastic cells (brown). All samples were counterstained with Eosin. Scale bar 10μm.

To investigate the expression of HCMV RNA and protein in the tumors we performed *in situ* hybridization (ISH), using a probe for the early beta 2.7 transcript and immunohistochemistry (IHC), using an antibody that recognizes a HCMV nuclear protein. The ISH and IHC detection received scores from 0–12, calculated by positive stained cells multiplied by staining intensity. A score of 4 was considered the cutoff for positivity. IHC immunoreactivity was found in 30 of 52 (57%) of the samples and in one non-tumoral brain sample (Fig 1B). Photomicrographs of representative tumoral cases and their scores are shown in Fig 2A. In all cases protein staining was found exclusively in the nucleus oftumoralcells. No positivity was found in non- non-neoplastic cells, such as endothelial cells and inflammatory cells.

**Fig 3:**
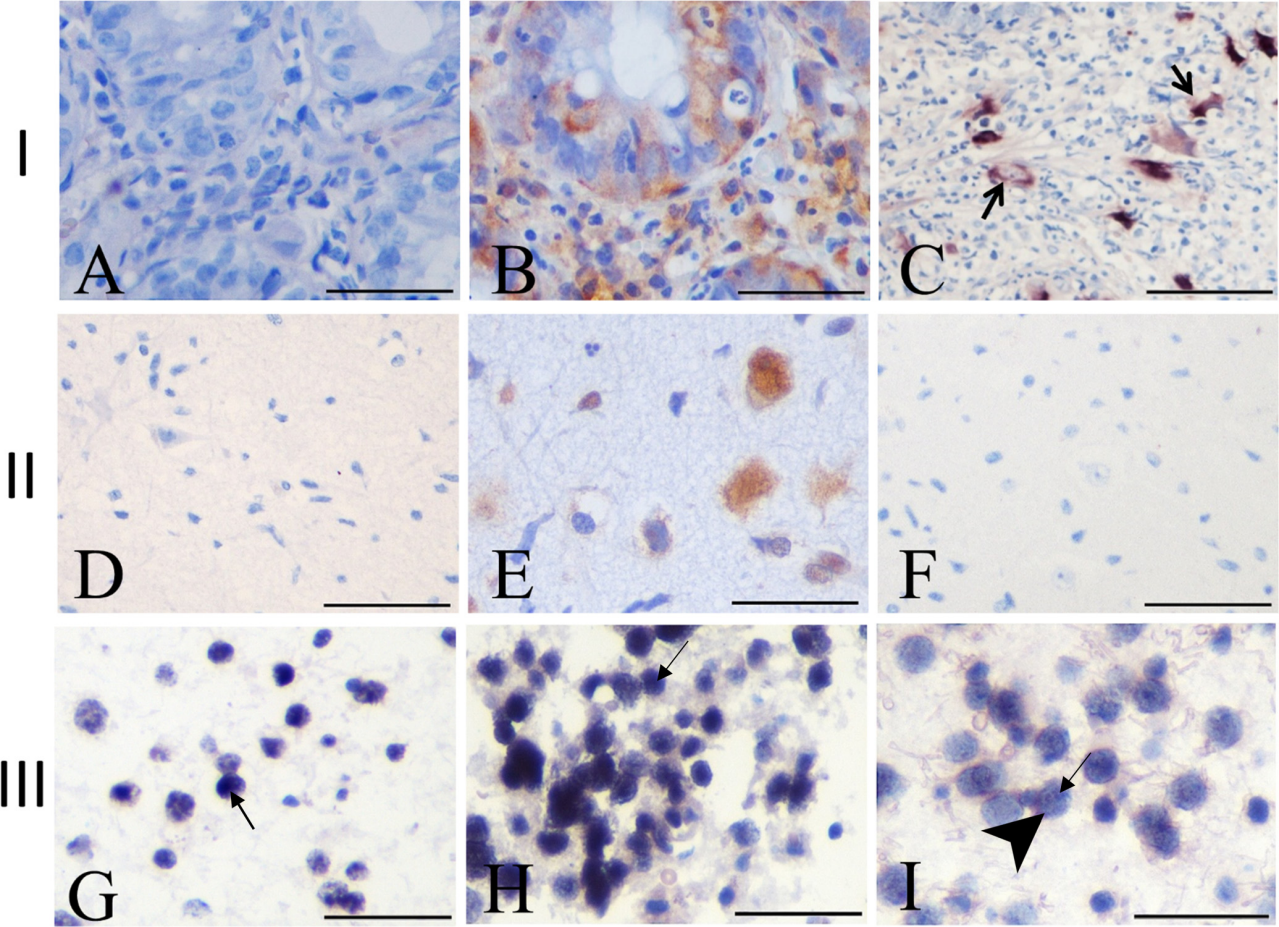
*Insitu* hybridization in gliomas. Row I: Infected HCMV colon tissue. (A) No probe. (B) Polyadenylic RNA probe. (C) HCMV 2.7 RNA probe. Row II: Non-tumoral brain. (D) No probe. (E) Polyadenylic RNA probe. (F) HCMV 2.7 RNA probe. Row III: Positive tumor samples. (G) AST I, score 4. (H) ODG II, score 12. (I) AST II, score 6. Arrows indicate nuclear signal in neoplastic cells (dark blue). In I, arrowhead and arrow indicate cytoplasmic and nuclear signal (blue). All samples were counterstained with Eosin. Scale bar = 10μm.

The early 2.7 RNA was detected by ISH in 19 of 52 (36%) of the cases (Fig 1C). Fig 3 shows cases of ODG II, AST I and II and their respective scores. None of the non-tumoral brain tissues were positive. No signal was observed by omission of the probe (Fig 3A and 3D). A probe specific for polyadenylic mRNA demonstrates the integrity of the RNA (Fig 3B and 3E). Hybridization signal was predominantly nuclear in (Fig 3C) tumor samples (Fig 3G and 3H). In positive control and in only one case of AST II (Fig 3I), RNA was found in similar levels in the cytoplasm and nucleus.

### Correlation between the HCMV presence and tumor grade

To verify a possible correlation between the presence of viral RNA and protein with tumor type or grade we compared IHC and ISH scores with each tumor type.

**Fig 4:**
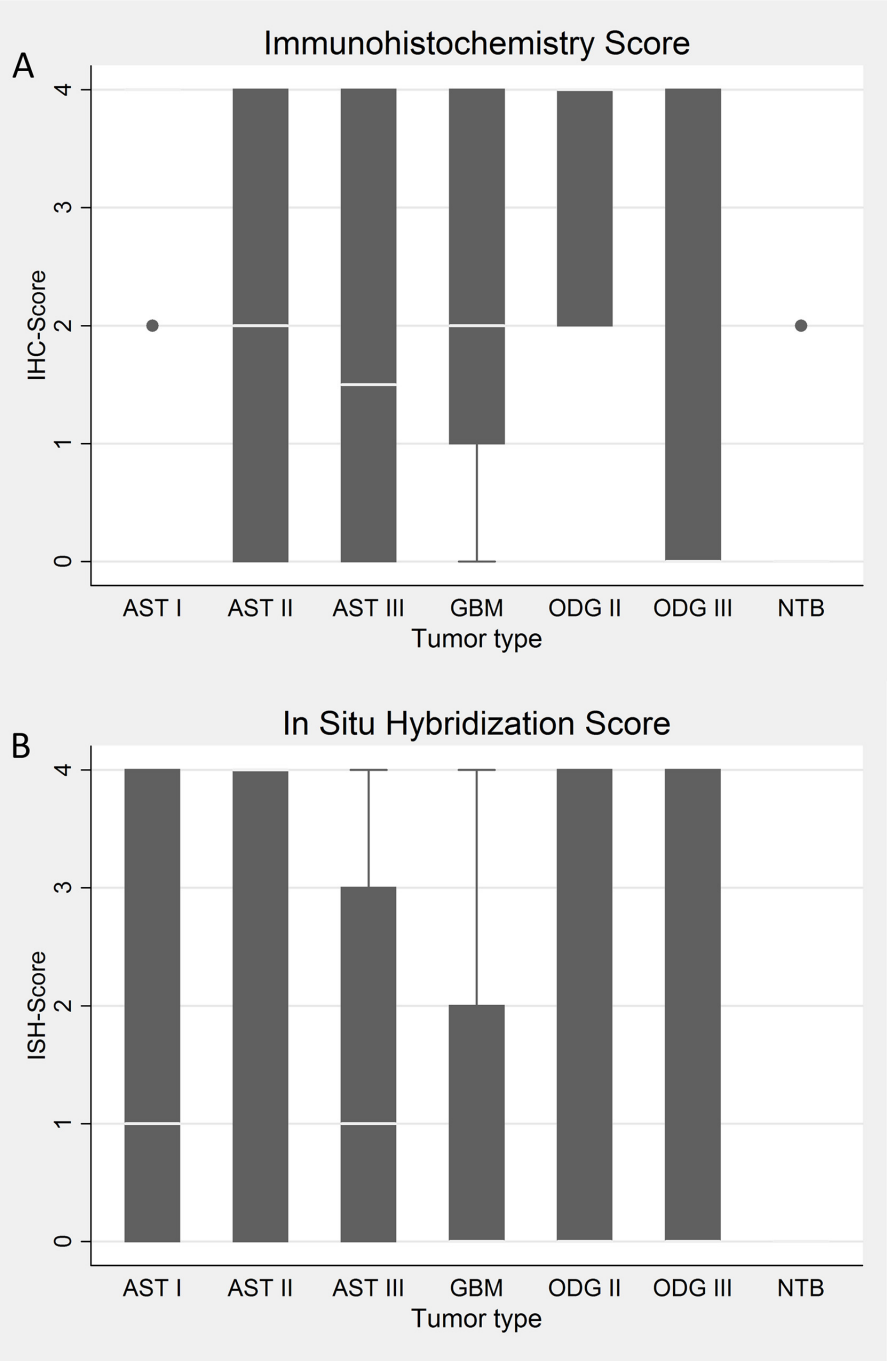
Immunohistochemistry and *in situ* hybridization scores in gliomas. IHC (A) and ISH (B) scores for astrocytomas (AST), oligodendrogliomas (ODG) and non-tumoral brain (NTB). p-values were 0.0014 and 0.0366, respectively.

All tumor types demonstrated heterogeneity in the number of positive cells and staining. Fig 4A and 4B shows IHC and ISH scores for the different tumor types. The median scores by IHC do not show any considerable variation, with exception of AST I, which presents a high score due to cell intensity in two samples, not considered statistically significant. These results indicate that there is no relationship between RNA and protein expression with tumor type or grade.

**Fig 5:**
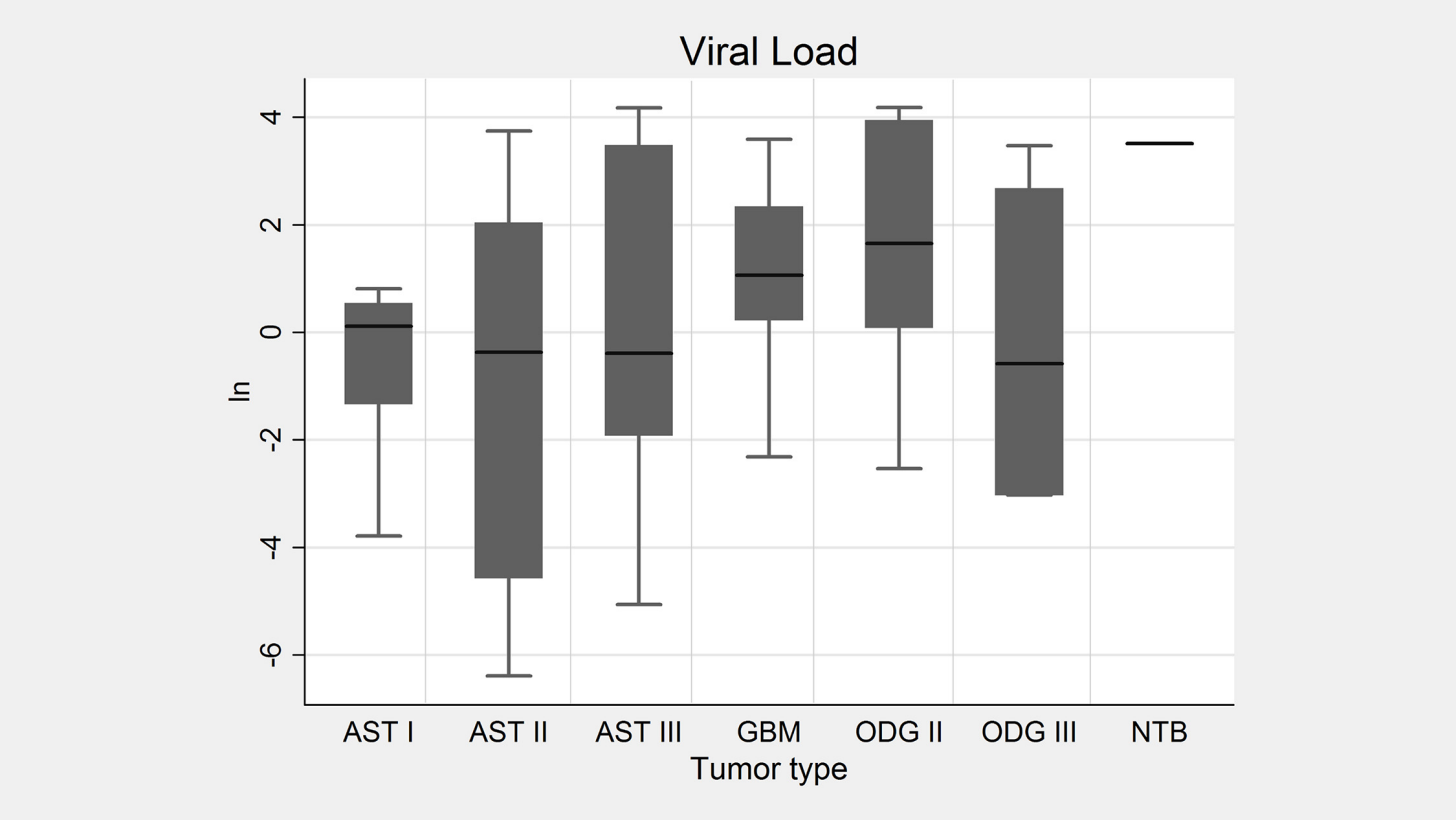
Viral load in gliomas. Box plot of the viral load for astrocytomas (AST), oligodendrogliomas (ODG) and non tumoral brain (NTB), shown as natural logarithm. p-value = 0.0102.

To further investigate if there is an association between viral DNA copy number and tumor progression we determined the viral load in the tumors. The number of viral DNA molecules per cells, as measured by qPCR, varied from 1,67x10^-5^ to 6,55x10^-1^ in the total tumors. A high intra tumor viral load heterogeneity was also observed (Fig 5 and S2 Table). The minimum, maximum and median viral load for each tumor type, as the respective converted natural logarithm (ln), demonstrated in Fig 5, show no significant difference in the viral DNA amount inter tumor types.

Finally, no parallel between viral load and tumor scores was observed.

## Discussion

The association of HCMV with gliomas has been a topic greatly debated in the brain tumor field. Increasing evidences demonstrate that HCMV is present in gliomas, especially in GBM, the most malignant type. Nevertheless, there are reports that failed to detect the virus in the tumors (S1 Table).

Here we show the presence of viral DNA, RNA and protein in fifty-two gliomas of different grades of malignancy.

By qPCR the UL83 viral region was detected in 38 of 52 (73%) of the total tumors. The highest frequency was observed in GBM (90%), followed by AST III (87%), AST I (71%), ODG II (66%), AST II (63%), ODG III (57%) (Fig 1A). The high positivity in GBM is consistent with our previous findings, were viral DNA was detected in 95% of fresh GBM tissues, using the same technique and the same set of primers [32]. Although the DNA was detected in the majority of the tumors, viral load analysis showed a low copy number, with an average of 1 copy in each 10 cells, consistent with previously reports [30,35].

The beta 2.7 RNA, the most abundantly transcribed early gene from the HCMV genome, in permissive cells [49], was detected at lower frequencies than viral DNA, 19 of 52 (36%) in the tumors by ISH (Fig 1C). In the majority of the samples, hybridization signal was found in the nucleus of tumor cells (Fig 3G and 3H), while signal was observed both in the nucleus and cytoplasm in the control positive colon tissue (Fig 3C). Wu et al, 1992, demonstrated that in permissive fibroblasts the beta 2.7 is localized both in the nucleus and cytoplasm, in a high proportion of the cells, whereas in non-permissive mouse fibroblasts the transcript is detected only in the nucleus in a lower number of cells. Our findings in glioma tissues show similar transcript localization of non-permissive cells. In fact HCMV existence in glioma cells does not fit in the concept of viral lytic or latent state. Although viral DNA and gene expression occurs in the tumors, only rare particles morphological consistent with HCMV were found in two GBM tissues by Cobbs et al, 2002.

Viral protein was detected in our study in 30 of 52 (57%) of the tumors. Positivity was higher in low grade gliomas (Fig 1B). Viral protein localization wasnuclear in all cases (Fig 2B, 2C and 2D) and only present in tumor cells.

Although the overall DNA frequency was higher, in some cases protein or RNA were detected in absence of viral DNA (S2 Table). This fact has been previously described by Mitchell et al and Ding et al [24,48].

A reason for this discrepancy could be the fact that the viral genome is not complete in tumors samples. However Ranganathan et al, demonstrated by PCR that multiple viral genomic regions are present in GBM samples, suggesting that the whole HCMV genome is present in the vast majority of the samples [28].

An additional possibility is that normal brain and necrotic tissue, not infected with the virus, are included in some specimens analyzed, resulting in missing tumor infected tissues when searching for viral DNA by PCR.

Another explanation is that HCMV genomic regions are lost after cellular transformation, presumably because continued presence of mutagenic viral gene products would be detrimental to cell survival, as proposed by the hit and run theory [50]. However, if the hit and run effect exists we expect to detect protein or RNA, in absence of DNA in a larger number of samples than we found (13 of 52).

Importantly, we show that of the 13 non-tumoral brain samples, derived from epileptic patients, one was positive by qPCR and one by IHC (cases 43 and 9 respectively—S2 Table). Few of the studies that analyzed the presence of the virus in gliomas, searched for the virus in non-tumoral brain samples. While Cobbs et al, Matlaf et al and Ding et al,did not detected the virus [21,33,48], Ranganathan et al and Mohammad et al, reported positive cases by PCR [28,35].

These findings support the theory that, after primary infection, the virus can be present in a latent or persistent stage in the central nervous system, with low levels of viral replication or gene expression that can barely be detected in non-tumoral tissues. In the tumor context the virus can reactivate and can consequently exert its oncomodulatory properties.

An important issue regarding the association of HCMV with gliomas is the correlation between the amount of virus with tumor malignancy.

Despite the limitation of a small study sample, our work suggests that there is no association between the presence of RNA and protein or viral load with tumor type and grade (Figs 4A, 4B and 5), in agreementwith the findings in previous studies [20,30,36,37,48].

Interestingly, two studies show that GBM patients with low grade of HCMV infection in the tumors survive longer than patients with high-grade infection[29,34].

It is possible that the virus is involved with tumor malignancy and consequently with patient survival with high grade tumors (GBM).

Unfortunately, we did not have access to patient survival data to investigate if the presence of HCMV infection is related to survival rate in this high malignant tumor.

Despite of all the findings reported in the literature so far at this stage is not determined if the virus has a role in glioma pathogenesis and further studies need to be done to demonstrate if the HCMV has indeed oncomodulatory properties. Regardless the possible role of the virus in tumorigenesis its simple presence in the tumors can be exploited as a target in tumor therapies.

## Supporting Information

S1 Table Published reports of HCMV detection in gliomas. (XLSX)

S2 Table Percentage of positive cells (%), staining intensity (int), final scores, obtained by IHC and ISH, and viral load in astrocytomas (AST), oligodendrogliomas (ODG) and non-tumoral brain (NTB). (DOCX)
