# Supplementary material for: Human Cytomegalovirus DNA Quantification and Gene Expression in Gliomas of Different Grades
Source: PLoS One. 2016 Jul 26;11(7):e0159604. doi: 10.1371/journal.pone.0159604 (PMC4961403; doi:10.1371/journal.pone.0159604)
Supplement: S2 Table — (DOCX) [file pone.0159604.s002.docx]

| Patient | Tumor Type | IHCp | IHCi | IHCscore | ISHp | ISHi | ISHscore | Viral Load |
| --- | --- | --- | --- | --- | --- | --- | --- | --- |
| 3 | NTB | 1 | 1 | 1 | 1 | 0 | 0 | 0,00E+00 |
| 9 | NTB | 4 | 1 | 4 | 1 | 0 | 0 | 0,00E+00 |
| 12 | NTB | 2 | 1 | 2 | 1 | 0 | 0 | 0,00E+00 |
| 15 | NTB | 1 | 1 | 1 | 1 | 0 | 0 | 0,00E+00 |
| 21 | NTB | 1 | 1 | 1 | 1 | 0 | 0 | 0,00E+00 |
| 28 | NTB | 1 | 1 | 1 | 1 | 0 | 0 | 0,00E+00 |
| 35 | NTB | 2 | 1 | 2 | 1 | 0 | 0 | 0,00E+00 |
| 38 | NTB | 1 | 0 | 0 | 1 | 0 | 0 | 0,00E+00 |
| 43 | NTB | 1 | 0 | 0 | 1 | 0 | 0 | 3,35E-01 |
| 46 | NTB | 1 | 0 | 0 | 1 | 0 | 0 | 0,00E+00 |
| 49 | NTB | 1 | 0 | 0 | 1 | 0 | 0 | 0,00E+00 |
| 59 | NTB | 1 | 1 | 1 | 1 | 0 | 0 | 0,00E+00 |
| 65 | NTB | 1 | 1 | 1 | 1 | 0 | 0 | 0,00E+00 |
| 4 | AST I | 4 | 2 | 8 | 1 | 1 | 1 | 1,12E-02 |
| 8 | AST I | 4 | 1 | 4 | 1 | 3 | 3 | 2,24E-02 |
| 25 | AST I | 4 | 3 | 12 | 1 | 1 | 1 | 1,67E-02 |
| 39 | AST I | 3 | 2 | 6 | 1 | 0 | 0 | 2,68E-03 |
| 44 | AST I | 4 | 2 | 8 | 4 | 2 | 8 | 0,00E+00 |
| 55 | AST I | 4 | 3 | 12 | 4 | 2 | 8 | 0,00E+00 |
| 58 | AST I | 4 | 2 | 8 | 1 | 0 | 0 | 2,26E-04 |
| 2 | AST II | 2 | 1 | 2 | 3 | 3 | 9 | 0,00E+00 |
| 17 | AST II | 1 | 0 | 0 | 2 | 3 | 6 | 1,05E-04 |
| 19 | AST II | 3 | 1 | 3 | 1 | 0 | 0 | 0,00E+00 |
| 27 | AST II | 4 | 3 | 12 | 3 | 2 | 6 | 7,46E-02 |
| 34 | AST II | 1 | 0 | 0 | 1 | 0 | 0 | 4,22E-01 |
| 36 | AST II | 2 | 1 | 2 | 2 | 2 | 4 | 3,24E-02 |
| 45 | AST II | 2 | 2 | 4 | 4 | 3 | 12 | 0,00E+00 |
| 50 | AST II | 3 | 2 | 6 | 2 | 2 | 4 | 6,16E-03 |
| 57 | AST II | 4 | 3 | 12 | 4 | 2 | 8 | 1,67E-05 |
| 60 | AST II | 3 | 1 | 3 | 1 | 0 | 0 | 6,90E-03 |
| 62 | AST II | 3 | 2 | 6 | 1 | 0 | 0 | 0,00E+00 |
| 1 | AST III | 1 | 0 | 0 | 1 | 2 | 2 | 6,52E-01 |
| 10 | AST III | 4 | 2 | 8 | 1 | 0 | 0 | 3,15E-01 |
| 14 | AST III | 2 | 2 | 4 | 1 | 2 | 2 | 1,50E-03 |
| 20 | AST III | 4 | 2 | 8 | 1 | 0 | 0 | 5,23E-02 |
| 22 | AST III | 1 | 1 | 1 | 1 | 0 | 0 | 6,74E-03 |
| 33 | AST III | 4 | 2 | 8 | 1 | 0 | 0 | 6,33E-05 |
| 42 | AST III | 3 | 1 | 3 | 4 | 3 | 12 | 0,00E+00 |
| 51 | AST III | 1 | 0 | 0 | 4 | 3 | 12 | 4,86E-03 |
| 6 | GBM | 3 | 2 | 6 | 1 | 0 | 0 | 2,69E-02 |
| 11 | GBM | 3 | 1 | 3 | 3 | 3 | 9 | 9,85E-04 |
| 18 | GBM | 3 | 2 | 6 | 1 | 2 | 2 | 0,00E+00 |
| 24 | GBM | 1 | 0 | 0 | 1 | 0 | 0 | 1,01E-01 |
| 30 | GBM | 2 | 1 | 2 | 3 | 3 | 9 | 9,89E-02 |
| 40 | GBM | 1 | 0 | 0 | 1 | 0 | 0 | 4,56E-03 |
| 47 | GBM | 3 | 2 | 6 | 1 | 0 | 0 | 1,28E-02 |
| 53 | GBM | 2 | 1 | 2 | 1 | 0 | 0 | 1,26E-01 |
| 56 | GBM | 3 | 2 | 6 | 1 | 0 | 0 | 3,63E-01 |
| 63 | GBM | 1 | 1 | 1 | 1 | 0 | 0 | 2,89E-02 |
| 5 | ODG II | 4 | 1 | 4 | 1 | 0 | 0 | 1,01E-01 |
| 13 | ODG II | 4 | 2 | 8 | 2 | 2 | 4 | 7,93E-04 |
| 16 | ODG II | 2 | 1 | 2 | 2 | 3 | 6 | 1,11E-02 |
| 26 | ODG II | 4 | 3 | 12 | 1 | 0 | 0 | 5,03E-01 |
| 29 | ODG II | 3 | 2 | 6 | 3 | 3 | 9 | 6,55E-01 |
| 41 | ODG II | 3 | 2 | 6 | 1 | 0 | 0 | 0,00E+00 |
| 54 | ODG II | 4 | 3 | 12 | 3 | 2 | 6 | 0,00E+00 |
| 61 | ODG II | 4 | 1 | 4 | 1 | 0 | 0 | 0,00E+00 |
| 64 | ODG II | 3 | 1 | 3 | 1 | 0 | 0 | 2,70E-02 |
| 7 | ODG III | 4 | 3 | 12 | 1 | 0 | 0 | 5,01E-04 |
| 23 | ODG III | 1 | 0 | 0 | 1 | 0 | 0 | 3,22E-01 |
| 31 | ODG III | 1 | 0 | 0 | 1 | 0 | 0 | 6,24E-02 |
| 32 | ODG III | 1 | 0 | 0 | 2 | 3 | 6 | 0,00E+00 |
| 37 | ODG III | 1 | 0 | 0 | 1 | 0 | 0 | 4,85E-04 |
| 48 | ODG III | 3 | 2 | 6 | 3 | 2 | 6 | 0,00E+00 |
| 52 | ODG III | 3 | 2 | 6 | 1 | 0 | 0 | 0,00E+00 |
